# Ameliorative Effect of *Posidonia oceanica* on High Glucose-Related Stress in Human Hepatoma HepG2 Cells

**DOI:** 10.3390/ijms24065203

**Published:** 2023-03-08

**Authors:** Marzia Vasarri, Emanuela Barletta, Maria Stio, Maria Camilla Bergonzi, Andrea Galli, Donatella Degl’Innocenti

**Affiliations:** 1 Department of Experimental and Clinical Biomedical Sciences, University of Florence, Viale Morgagni 50, 50134 Florence, Italy; 2 Department of Chemistry “Ugo Schiff”, University of Florence, Via Ugo Schiff 6, 50019 Sesto Fiorentino, Florence, Italy; 3 Interuniversity Center of Marine Biology and Applied Ecology “G. Bacci” (CIBM), Viale N. Sauro 4, 57128 Livorno, Italy

**Keywords:** lipid accumulation, high glucose, NF-κB, MMP-2/9, HepG2 cells, *Posidonia oceanica*, marine products

## Abstract

Metabolic disorders characterized by elevated blood glucose levels are a recognized risk factor for hepatocellular carcinoma (HCC). Lipid dysregulation is critically involved in the HCC progression, regulating energy storage, metabolism, and cell signaling. There is a clear link between de novo lipogenesis in the liver and activation of the NF-κB pathway, which is involved in cancer metastasis via regulation of metalloproteinases MMP-2/9. As conventional therapies for HCC reach their limits, new effective and safe drugs need to be found for the prevention and/or adjuvant therapy of HCC. The marine plant *Posidonia oceanica* (L.) Delile is endemic to the Mediterranean and has traditionally been used to treat diabetes and other health disorders. The phenol-rich leaf extract of *Posidonia oceanica* (POE) is known to have cell-safe bioactivities. Here, high glucose (HG) conditions were used to study lipid accumulation and fatty acid synthase (FASN) expression in human HepG2 hepatoma cells using Oil Red O and Western blot assays. Under HG conditions, the activation status of MAPKs/NF-κB axis and MMP-2/9 activity were determined by Western blot and gelatin zymography assays. The potential ameliorative role of POE against HG-related stress in HepG2 cells was then investigated. POE reduced lipid accumulation and FASN expression with an impact on de novo lipogenesis. Moreover, POE inhibited the MAPKs/NF-κB axis and, consequently, MMP-2/9 activity. Overall, these results suggest that *P. oceanica* may be a potential weapon in the HCC additional treatment.

## 1. Introduction

Hepatocellular carcinoma (HCC) is the second leading cause of cancer death worldwide and remains a global health challenge [1]. HCC can have various etiologic causes, but among the most important risk factors for HCC are metabolic disorders characterized by elevated blood glucose levels, including diabetes, obesity, and metabolic syndrome. The alarming increase in these metabolic disorders worldwide reflects the rising incidence of HCC [2,3]. Although the mechanisms by which obesity and steatosis promote liver carcinogenesis remain quite unclear, the role of lipid dysregulation in this process is widely recognized [4]. Lipid dysregulation is critical in both the development and progression of liver cancer [5]. There is ample evidence that lipids promote tumor activity by controlling various biological processes, including energy storage and metabolism, epigenetic regulation, cell signaling, and many others [6].

De novo lipogenesis represents one of the hallmarks of cancer and is frequently upregulated in solid tumors, reducing cancer cell growth’s reliance on exogenous fatty acids [7].

Increased expression of fatty acid synthase (FASN), which catalyzes de novo synthesis of long-chain fatty acids, has been described in several tumor types, while its inhibition has been shown to have antitumor activity [8].

Recent literature reports a correlation between hepatic lipogenesis and nuclear factor kappa B (NF-κB) activation [9]. As a matter of fact, NF-κB is one of the major transcription factors that regulate development, inflammatory responses, and tackle nutritional stress (high carbohydrate diet/high-fat diet) by fostering lipogenic stimulus [9]. The NF-κB pathway activation is significantly associated with poor prognostic traits as well as stemness characteristics, which places modulation of NF-κB signaling in the focus of therapeutic interventions [10]. 

Stress-related pathways, such as the p38 mitogen-activated protein kinase (MAPK) signaling cascades, have been shown to contribute to the NF-κB response [11]. 

Furthermore, NF-κB signaling has been shown to contribute to cancer progression by controlling epithelial-mesenchymal transition and metastasis [12,13]. Indeed, NF-κB is one of the most important upstream regulators of matrix metalloproteinases, including MMP-2/9, which play a key role in HCC invasion and metastasis [14,15]. Therefore, targeting the NF-κB signaling pathway may be a promising strategy for the therapeutic management of HCC and the improvement of patient prognosis.

For years now, scientific research on marine natural products has been widespread, demonstrating how the sea can be instrumental in combating human diseases. Marine compounds have the potential for pharmacological activities, such as anticancer, antiviral, antioxidant, antimicrobial, anti-inflammatory, and many more [16,17].

The marine plant *Posidonia oceanica* (L.) Delile is the only member of the Posidoniaceae family that is endemic to the Mediterranean. *P. oceanica* leaves were used in folk medicine to treat a variety of human health issues [18], including skin problems and sore throats [19], irritation and inflammation, joint pain, and acne [20]. *P. oceanica* leaf decoction was also used to treat diabetes and hypertension [21].

For years, our research group has been studying the bioactive properties of a hydroalcoholic extract of *P. oceanica* leaves (POE) [18,22,23,24,25,26,27,28]. The phenolic composition of POE has been identified as 88% and is predominantly represented by (+) catechins, and minimally by gallic acid, ferulic acid, epicatechin, and chlorogenic acid (Table 1) [22].

POE has been shown to possess antioxidant and anti-inflammatory [23,24], anti-glycation properties [25], and to inhibit cancer cell migration [22,26,27]. Therefore, given the biological properties of POE, here, the potential role of POE under high glucose-related stress conditions in a cellular model of HCC was studied.

Several commonly used human HCC cell lines are reported in the literature [29]. However, HepG2 is the most widely used cell line and is generally considered a good model of liver cancer, including HCC [30].

Because metabolic disturbances due to high blood glucose levels can be risk factors for tumor progression, in this work, HepG2 human hepatoma cells were exposed to 25 mM D-Glucose (high glucose, HG), which is higher than physiological glucose (normal glucose, NG, 5 mM), to study HG-related stress, an experimental model already described in the literature [31,32,33].

Specifically, lipid accumulation, MAPKs/NF-κB axis activation status, and MMP-2/9 activity in HepG2 cells were evaluated under HG conditions. 

Considering the described bioactivities of POE, the potential ameliorative role of the phytocomplex against high glucose-related stress in HepG2 cells was then evaluated.

## 2. Results and Discussion

### 2.1. Extraction Yield from P. oceanica Leaves and Its Biochemical Properties

The hydroalcoholic extraction protocol, previously described [26,34], was applied to 4 g of dried and minced leaves of *P. oceanica*. The total yield of *P. oceanica* dry extract was 45 mg. POE was constituted by resuspending 1.8 mg of dry extract in 0.5 mL of EtOH 70% (*v*/*v*), yielding a hydrophilic analyte concentration of 3.6 mg/mL.

The Folin–Ciocalteau assay was used to determine the total polyphenol (TP) content of POE. Its antioxidant and radical scavenging properties were analyzed by Ferric Reducing Antioxidant Power (FRAP) and DPPH colorimetric assays. TP content of POE was found to be 3.6 ± 0.3 mg/mL of gallic acid equivalents (GAE), while its antioxidant and radical scavenging activity were found to be 1.0 ± 0.2 and 10 ± 2.0 mg/mL of ascorbic acid equivalents (AAE), respectively. Data of POE biochemical characterization are reported as mean ± standard deviation (SD) of three independent experiments (Table 2).

Accordingly, these results support the efficacy and reproducibility of hydroalcoholic extractions from *P. oceanica* leaves [26,27,34,35].

As in a previous work [30], the non-cytotoxic 7 µg GAE/mL dose of POE was used for subsequent cell-based experiments.

### 2.2. Effect of POE on Lipid Accumulation under High Glucose Condition in HepG2 Cells

High levels of glucose in the bloodstream are known to cause cell toxicity, resulting in cellular damage and organ dysfunction [36]. However, in cancer cells, the enormous energy demand for rapid proliferation and expansion is mainly provided by glucose utilization. Indeed, aerobic glycolysis is a distinctive process in many cancers, including HCC, and regulates tumor progression [37,38]. 

**Figure 1:**
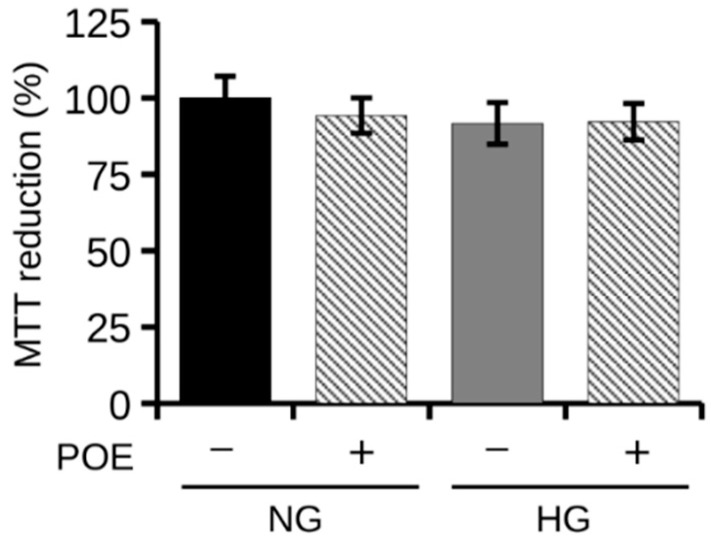
Viability of HepG2 cells under normal (NG, 5 mM D-glucose) or high (HG, 25 mM D-glucose) glucose conditions in the absence (−) or presence (+) of POE (7 µg GAE/mL) for 24 h. Cell viability was assessed by MTT assay. Values are represented as mean ± SD of three independent experiments. Values are reported as percentages compared with untreated control cells under NG conditions.

To evaluate the effect of high glucose (HG) concentrations on the viability of HepG2 human hepatoma cells, cells were exposed to normal glucose (NG, 5 mM D-glucose) or high glucose (HG, 25 mM D-glucose) conditions for 24 h. However, as shown in Figure 1, the exposure for 24 h to HG had no significant effect on cell viability, which remained comparable to that of cells under NG conditions. In addition, the presence of POE (7 μg GAE/mL) did not affect cell viability under both NG and HG conditions (Figure 1).

Lipid metabolism is now recognized as an important pathway in cancer. It may provide additional energy sources needed for metastasis, assembly blocks for proliferation, and act as secondary messengers in various signaling pathways [39].

**Figure 2:**
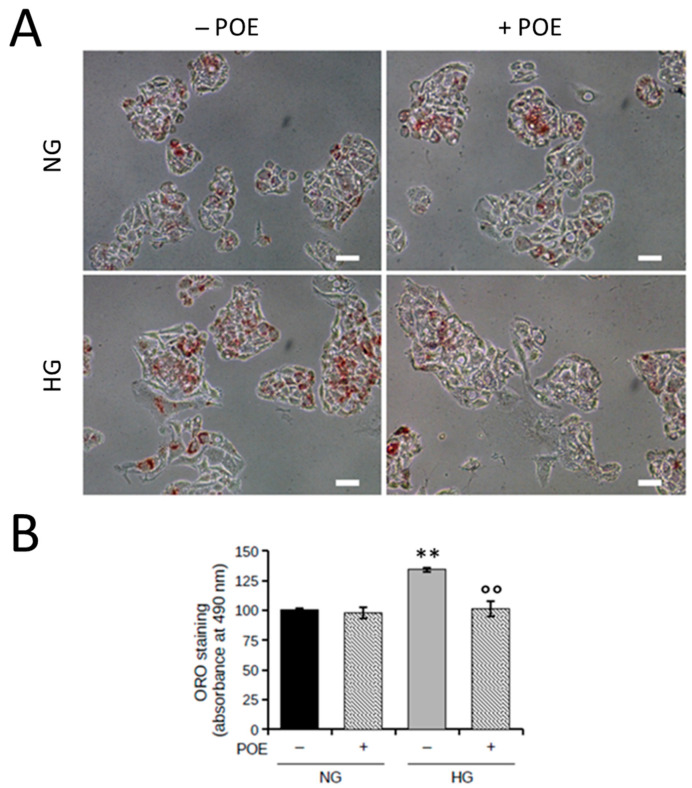
Intracellular lipid accumulation in HepG2 cells under normal glucose (NG, 5 mM) or high glucose (HG, 25 mM) conditions in the absence or presence of POE (7 µg GAE/mL) after 24 h treatment. (**A**) Representative image of ORO staining. Scale bar = 100 µm. (**B**) Changes in intracellular lipid content assessed by measuring ORO absorbance at 490 nm. Data were reported as the mean ± SD of independent experiments. ** *p* < 0.01 vs. NG untreated control cells; °° *p* < 0.01 vs. HG untreated control cells. Tukey’s HSD test.

The literature reports that high glucose levels result in increased intracellular lipid accumulation in HepG2 cells over 24 h [33]. To evaluate the effect of HG on intracellular lipid accumulation, HepG2 cells were exposed to HG for 24 h, and total neutral lipids were estimated by Oil Red O (ORO) staining (Figure 2A). As expected, high concentrations of glucose (25 mM) resulted in a significant increase in neutral lipid accumulation of approximately 35% (134 ± 2%) in HepG2 cells compared with control cells exposed to physiological concentrations of glucose (5 mM) (Figure 2B).

Hence, the ability of POE to prevent HG-induced lipid accumulation in HepG2 cells was evaluated. As shown in Figure 2A, the high levels of HG-induced intracellular neutral lipids were significantly reduced in the presence of POE (101 ± 6%), which is comparable to those of control cells under NG conditions (Figure 2B). These data demonstrated the ability of POE to inhibit lipid accumulation induced by high glucose concentrations.

### 2.3. Role of High Glucose on FASN Expression in HepG2 Cells and the Effect of POE

The high aerobic metabolism in HCC is accompanied by an activated glycolytic flux resulting in increased metabolic intermediates. These intermediates can be used for the biosynthesis of macromolecules, including triglycerides and phospholipids, to meet the demands of rapid tumor growth [40]. When glucose enters the cell by specific transporters, it is converted to pyruvate by glycolysis and then to acetyl-CoA to enter the Krebs cycle. In the presence of excess glucose, citrate from the Krebs cycle is exported to the cytoplasm. Citrate is the main inducer of acetyl-CoA carboxylase activity that produces malonyl-CoA, the major intermediate in fatty acid synthesis. A key role during this process is played by fatty acid synthase (FASN), which consumes acetyl-CoA and malonyl-CoA by catalyzing the de novo synthesis of fatty acids [41]. FASN plays an essential role in the lipid metabolic pathway and can rewire tumor cells to have greater energy flexibility to meet their high energy demands. As a consequence, FASN plays a crucial role in the production of lipids in the liver, which can then be exported to metabolically active tissues or stored in adipose tissue [42]. Some cancer cells, including HCC cells, exhibit high FASN expression and promote the endogenous synthesis of fatty acids, providing energy for their proliferation [43].

**Figure 3:**
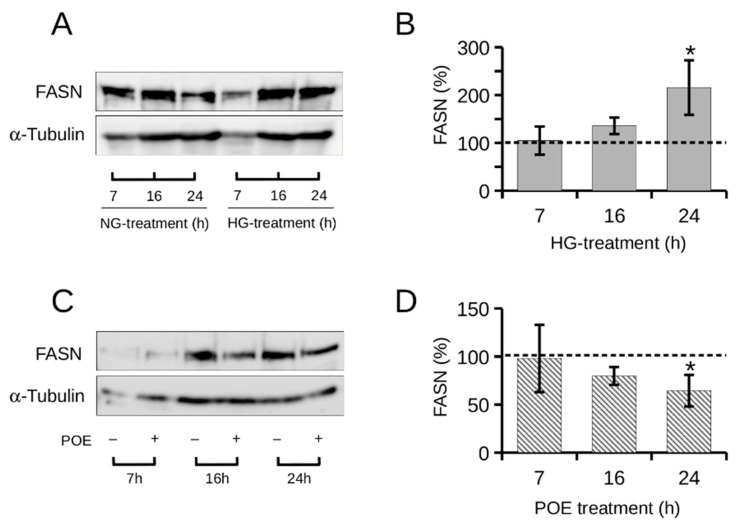
Representative images of FASN in HepG2 cells over time by Western blot assay (**A**) under normal glucose (NG, 5 mM) or high glucose (HG, 25 mM) conditions, and (**C**) in the absence or presence of POE (7 µg GAE/mL) under HG conditions. α-Tubulin (55 kDa) was used as a housekeeping protein in all expression analyses and as the loading control (**B**,**D**) Signal quantification was determined by densitometric analysis and reported as the mean of independent experiments. Error bars represent standard errors. Statistical analysis was performed by Kruskal–Wallis test: * *p* < 0.05 vs. (**B**) untreated NG control cells (represented by the dashed line), or (**D**) untreated HG control cells (represented by the dashed line).

Here, FASN expression levels were monitored in HepG2 cells during 24 h of HG exposure by Western blot analysis (Figure 3A). Under HG conditions, FASN expression levels increased progressively over time to an approximately twofold and significant increase (215 ± 56%) at 24 h exposure to HG treatment compared with NG treatment (Figure 3B). The expression levels of FASN in the intervals in the presence of physiological NG glucose (5 mM) did not change significantly over time (Figure S1 in Supplementary Materials).

These data agree with evidence in the literature showing an increase in hepatic FASN protein levels in response to increased glucose [44,45]. Indeed, FASN expression has also been shown to increase dramatically both in the liver of patients with the most severe degree of nonalcoholic fatty liver disease (NAFLD) and in mice with NAFLD induced by a high-fat diet [45]. 

Pharmacological inhibition of FASN has been shown to be effective in various malignant cells in vitro and in vivo but not in normal cells, and this represents a therapeutic window of intervention [39]. Therefore, the effect of POE on FASN expression in HepG2 cells exposed to HG was investigated (Figure 3C). As shown in Figure 3D, FASN levels were significantly reduced in POE-treated HG cells by about 35% (64 ± 15%) at 24 h of treatment compared with untreated HG cells, whereas POE showed no significant effect on FASN expression levels under physiological NG glucose conditions (Figure S2 in the Supplementary Materials).

These results are in agreement with data from the ORO assay and suggest that POE attenuates lipid accumulation induced by high glucose levels through down-regulation of the HG-related de novo lipogenesis.

### 2.4. Effect of High Glucose on NF-κB and MAPKs Signaling Pathways

Metabolic reprogramming critically influences cancer pathogenesis and progression. Lipid metabolism is an essential source of energy for cancer cells and plays an important role in microenvironment adaptation and cell signaling [4]. In this context, altered lipid metabolism could induce inflammation and promote fibrosis and support the progression of HCC [4,46].

Recent literature reports a correlation between hepatic de novo lipogenesis and NF-κB activation, particularly with a high-carbohydrate diet [9]. 

In addition, NF-κB signaling has been shown to be constantly activated in HCC tissues and cells [14]. 

**Figure 4:**
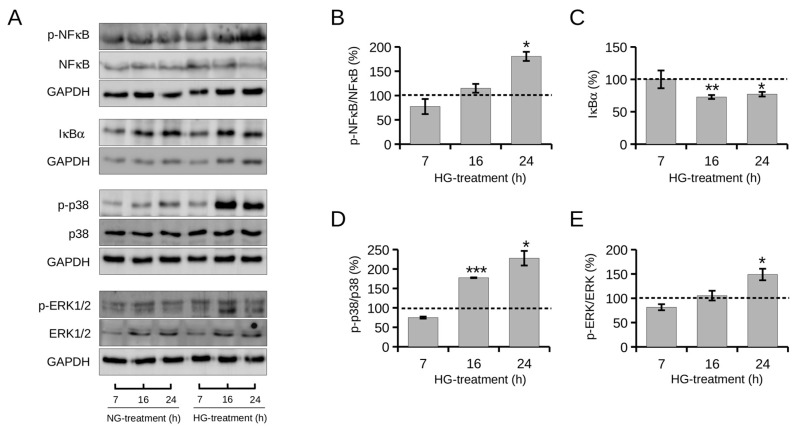
Expression levels over time of protein markers of NF-κB and MAPKs signaling pathways in HepG2 cells under normal (NG, 5 mM) or high (HG, 25 mM) glucose conditions. (**A**) Representative image of Western blot analysis. GAPDH (37 kDa) was used as a housekeeping protein in all expression analyses and as the loading control. Quantification of the signals of (**B**) p-NF-κB versus total NF-κB protein, (**C**) IκBα, (**D**) p-p38 versus total p38 protein, and (**E**) p-ERK1/2 versus total ERK1/2 protein was determined by densitometric analysis. Data were reported as the mean of independent experiments and in terms of percentages compared with control cells under NG conditions (represented by dashed line). Error bars represent standard errors. * *p* < 0.05, ** *p* < 0.01, *** *p* < 0.001 compared with control cells under NG conditions. Kruskal–Wallis test.

In this work, the activation status of the NF-κB signaling pathway under HG conditions was assessed by monitoring the expression levels of phosphorylated NF-κB transcription factor (p-NF-κB) and its cytosolic inhibitor IκBα by Western blot analysis (Figure 4A).

As shown in Figure 4B, in HG cells, the phosphorylation levels of NF-κB increased progressively over time to a significant increase of 80% at 24 h of treatment (180 ± 9%) compared with control cells under NG conditions. The progressive increase in p-NF-κB levels was matched by a progressive decrease in IκBα levels (Figure 4C). HG treatment resulted in a significant reduction in IκBα levels by approximately 27% from 16 h (72 ± 3%) compared with control cells under NG conditions. These data demonstrate that HG induces activation of the NF-κB signaling pathway, in agreement with the literature [47]. The expression levels of p-NF-κB/NF-κB and IκBα in the time intervals under NG conditions did not change significantly over time (Figure S3 in Supplementary Materials). Stress-related pathways, such as the MAPK (mitogen-activated protein kinase) signaling cascade, have been shown to contribute to the NF-κB response in the liver [10]. MAPK is composed of extracellular signal-regulated kinases (ERKs), c-Jun NH2-terminal kinases, and p38-MAPK (p38), contributing to inflammation, cell survival, and natural cell death [48]. The literature reports that several oncogenic kinases, including p38 and extracellular signal-regulated kinase (ERK) participate in the activation of NF-κB, contributing to the progression of HCC [10,14,49].

In light of these considerations, activation of the p38 MAPK signaling cascade in HepG2 cells under HG conditions was evaluated here by monitoring up to 24 h phosphorylation of some components of cell signaling, namely p-p38 and p-ERK1/2, by Western blot analysis (Figure 4A). 

Figure 4D shows a progressive increase in p-p38 levels over time. At 16 h of HG exposure, p-p38 levels were significantly increased by about 77% (177 ± 0.8%) compared with those of NG control cells, to reach an increase of about 127% (227 ± 18%) at 24 h. Comparably, HG resulted in a progressive increase in p-ERK1/2 levels that were significantly higher (148 ± 11%) than those of control cells in NG at 24 h of treatment. The expression levels of p-p38/p38 and p-ERK1/2/ERK1/2 in the time intervals under NG conditions did not change significantly over time (Figure S3 in Supplementary Materials).

Overall, these results indicate that HG stimulates phosphorylation of p38 and ERK1/2 in HepG2 cells, in agreement with the literature [31], suggesting the involvement of the MAPK signaling cascade in the NF-κB signaling response induced by high glucose levels.

### 2.5. Effect of POE on High Glucose-Induced NF-κB and MAPKs Signaling Pathways in HepG2 Cells

Because NF-κB activation plays a central role in cancer development, the NF-κB signaling pathway has been recognized as a potential therapeutic target in cancer. Some experimental evidence has demonstrated the ability of inhibitors of the MAPKs/NF-κB axis to block HCC progression [50,51]. Other evidence suggests that suppression of NF-κB/p65 gene transcription makes HepG2 cells chemosensitive [52] and that pharmacological inhibition of NF-κB attenuated hepatic lipid accumulation both in vitro and in vivo in response to a high carbohydrate diet [9].

**Figure 5:**
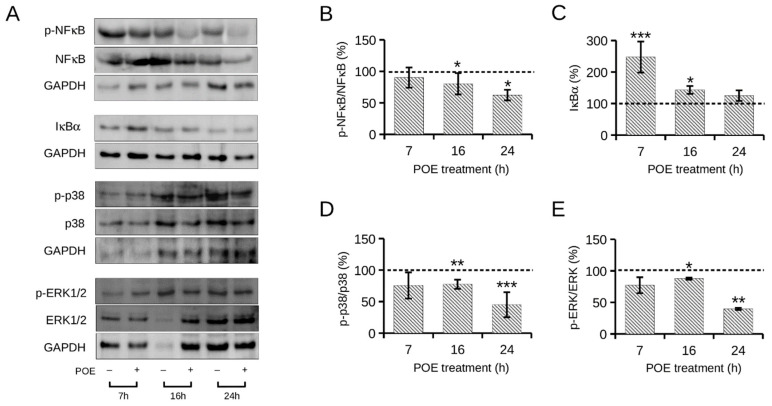
Expression levels over time of protein markers of NF-κB and MAPKs signaling pathways in HepG2 cells in the absence or presence of POE (7 µg GAE/mL) under high glucose conditions (HG, 25 mM). (**A**) Representative image of Western blot analysis. GAPDH (37 kDa) was used as a housekeeping protein in all expression analyses and as the loading control. Quantification of the signals of (**B**) p-NF-κB versus total NF-κB protein, (**C**) IκBα, (**D**) p-p38 versus total p38 protein, and (**E**) p-ERK1/2 versus total ERK1/2 protein was determined by densitometric analysis. Data were reported as the mean of independent experiments and in terms of percentages compared with control cells treated with HG in the absence of POE (represented by dashed line). Error bars represent standard errors. * *p* < 0.05, ** *p* < 0.01, *** *p* < 0.001 compared with untreated HG control cells. Kruskal–Wallis test.

In this work, the effect of POE on the activation status of NF-κB and MAPK signaling pathways induced by HG was evaluated by Western blot analysis (Figure 5A). As shown in Figure 5B, POE induced a slight but significant decrease in HG cells of about 30% in p-NF-κB levels as early as 16 h of treatment (70 ± 14%) compared with untreated HG cells. This inhibitory effect of POE was intensified at 24 h of treatment when p-NF-κB levels in HG-treated cells were about 40% lower (62 ± 8%) than in untreated HG cells. 

**Figure 6:**
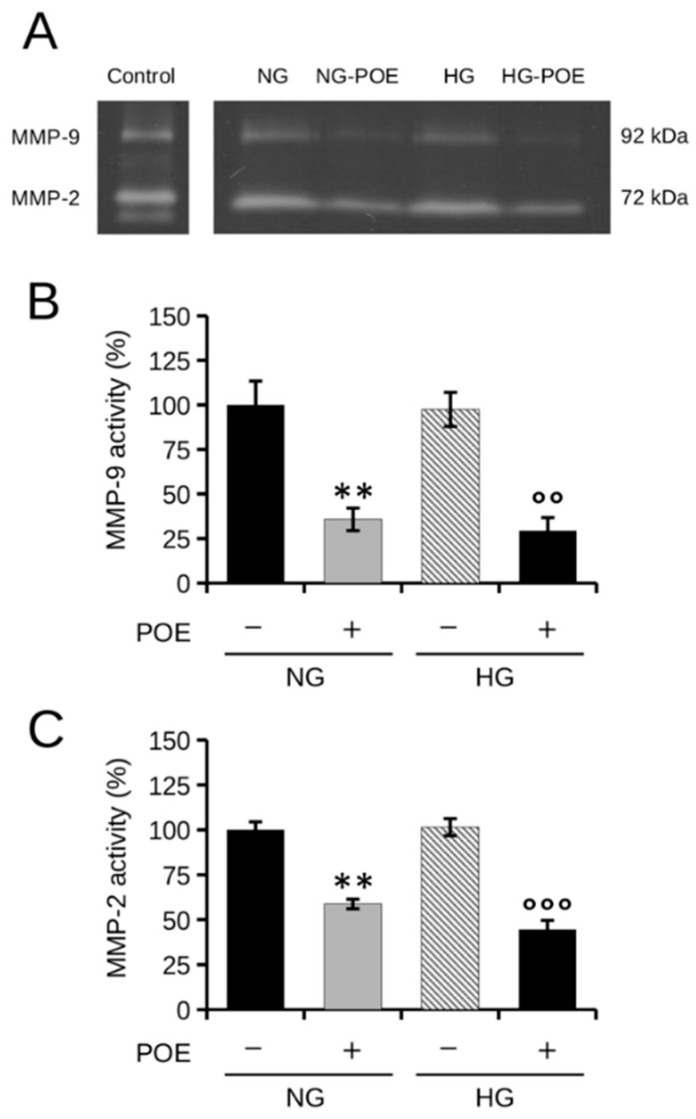
Effect of POE on the activity of MMP-2/9 released in culture medium. (**A**) Representative image of gelatin zymography of cell culture media collected at 24 h from HepG2 cells untreated (−) or treated with POE (7 μg GAE/mL) under normal glucose (NG, 5 mM D-glucose) and high glucose (HG, 25 mM D-glucose) conditions. Culture medium of untreated HT1080 human fibrosarcoma cells was used as a control for the molecular size of MMPs (Control). Quantitative data of gelatinolytic bands of (**B**) MMP-9 and (**C**) MMP-2. Data are reported as means ± SD from different experiments. Values are given as percentages compared with untreated control cells under NG conditions. ** *p* < 0.01 vs. NG control cells; °° *p* < 0.01, °°° *p* < 0.001 vs. HG control cells. Tukey’s HSD test.

The POE-induced inhibition of NF-κB activation was confirmed by the approximately 150% increase in IκBα levels (247 ± 49%) observed at 7 h of treatment in HG cells compared with untreated HG cells (Figure 5C). This suggests that POE prevents degradation of cytosolic IκBα at early times and that at later times, it prevents phosphorylation, and thus, activation of NF-κB, following a precise temporal pattern. In addition to NF-κB signaling, POE showed an influence on the MAPKs cascade. Specifically, as depicted in Figure 6D,E, both p-p38 and p-ERK1/2 were slightly, but significantly, reduced at 16 h of POE treatment in HG cells (77 ± 7% and 87 ± 1%, respectively) compared with untreated HG control cells. The inhibitory effect of POE on the activation of p38 and ERK1/2 was significantly more pronounced at 24 h of POE treatment in HG cells, when p-p38 and p-ERK1/2 were reduced by approximately 50% (45 ± 19% and 39 ± 1%, respectively) compared with untreated HG cells. The expression levels of these protein targets under NG conditions in the presence of POE did not change significantly over time (Figure S4 in Supplementary Materials). 

These findings are in line with the role of POE on the NF-κB signaling pathway previously demonstrated in lipopolysaccharide-stimulated murine macrophages [23]. 

This study is the first experimental evidence that POE is able to block HG-induced lipid accumulation in HepG2 cells by acting on the MAPKs/NF-κB axis. Several phytochemicals, particularly polyphenols, exert anticancer properties by targeting and inhibiting NF-κB and MAPK signaling pathways [53]. The action of POE could thus be attributed to the synergistic action of its phenolic constituents. 

### 2.6. Effect of POE on MMP-2/9 Activity in High Glucose Conditions in HepG2 Cells

HCC is one of the most lethal cancers, mainly because of its high tendency to metastasize. Metalloproteinases (MMPs) are key enzymes involved in extracellular matrix degradation. In particular, high levels of gelatinase MMP-2 and MMP-9 are known to correlate with invasion, metastasis, and poor prognosis in various types of cancer, including HCC [15]. In several human cancers, NF-κB has been observed to be one of the most important upstream regulators of MMPs, thus playing a key role in cancer development and progression [54]. 

Given the inhibitory role of POE on the NF-κB signaling pathway described above, the activity of MMP-2/9 was examined by gelatin zymography assay on the culture media of cells exposed to NG or HG in the absence or presence of POE for 24 h. 

Notably, POE resulted in a marked reduction of MMP-2/9 activity after 24 h of treatment of HepG2 cells in both NG and HG conditions (Figure 6A). Specifically, MMP-9 activity was reduced by 65% (35 ± 6%) and 70% (29 ± 7%) in POE-treated cells compared with untreated NG and HG control cells, respectively (Figure 6B), whereas POE treatment reduced MMP-2 activity by approximately 40% (58 ± 2%) and 55% (44 ± 5%) in HepG2 cells compared with untreated NG and HG control cells, respectively (Figure 6C). 

This evidence can be traced back to the ability of POE to inhibit the NF-κB signaling pathway. In fact, as described in the literature, blocking NF-κB signaling results in reduced invasiveness of HCC cells and reduced expression of invasion-related molecules, including MMP-2/9 [14,55]. 

## 3. Materials and Methods

### 3.1. Materials and Reagents

Dulbecco’s Modified Eagle’s Medium (DMEM, with high or low glucose), fetal bovine serum (FBS), L-glutamine, penicillin and streptomycin, 1-(4,5-dimethylthiazol-2-yl)-3,5-diphenyl formazan (MTT), 2,2-diphenyl-1-picrylhydrazyl (DPPH), 3-(2-pyridyl)-5,6-diphenyl-1,2,4-triazine-p,p′-disulfonic acid hydrate (Ferrozine^®^), Folin-Ciocalteau reagent, gallic acid, ascorbic acid, Oil red O solution, Coomassie Brilliant Blue G-250, gelatin, and all chemicals and solvents were purchased from Merck KGaA (Darmstadt, DA, Germany). Electrophoresis reagents were purchased from Bio-Rad Laboratories (Hercules, CA, USA). Primary antibodies were supplied by Cell Signaling Technology (Beverly, MA, USA), Molecular Probes^TM^ (Invitrogen, Carlsbad, CA, USA), and Santa Cruz (Heidelberg, Germany) (Table 3). HRP-linked anti-mouse IgG and anti-rabbit IgG secondary antibodies were obtained from Molecular Probes^TM^ (Invitrogen, Carlsbad, CA, USA). Disposable plastic was provided by Sarstedt (Milan, Italy).

### 3.2. Hydroalcoholic Extract from Leaves of P. oceanica: Preparation and Biochemical Characterization

Fresh leaves of *P. oceanica* L. Delile were collected in July and washed thoroughly with double-distilled water to remove surface epiphytes. 

The hydrophilic component was recovered according to the previously described protocol [26]. Briefly, 1 g of dried leaves of *P. oceanica* were crushed and suspended overnight in 10 mL of EtOH/H_2_O (70:30 *v*/*v*) at 37 °C under stirring and then at 65 °C for 3 h. The debris was then removed by centrifugation at 2000× *g*, and the recovered supernatant was mixed with *n*-hexane in a 1:1 ratio. 

The hydrophilic component of the extract was then recovered after repeated agitation in a separatory funnel, dispensed into 1 mL aliquots, and then dried using a Univapo^TM^ vacuum concentrator. 

An aliquot of *P. oceanica* leaf extract containing 1.8 mg of dry extract was dissolved in 0.5 mL of EtOH/H_2_O (70:30 *v*/*v*) before use and hereafter referred to as POE.

POE was then characterized for total polyphenol content (TP) by the Folin–Ciocalteau colorimetric method, as previously described [22,34]. Briefly, a solution of Folin–Ciocalteu’s phenol reagent (diluted 1:10 in distilled water) was added to scalar volumes of POE (final volume 20 µL) in a 96-well plate. After 5 min at room temperature, 80 µL of 7.5% sodium carbonate solution was added per well and incubated for 2 h. Gallic acid (0.5 mg/mL) was used as a reference in the range 0–10 µg to determine TP values. The absorption values were recorded with a microplate reader at 595 nm.

In addition, the antioxidant and radical-scavenging activities of POE were studied using the ferric-reductive/antioxidant power (FRAP) assay and the 2,2-diphenyl-1-picrylhydrazyl (DPPH) assay, respectively [22,34]. 

Briefly, for FRAP assay, distilled water was added to graduated volumes of POE to a final volume of 50, and 200 µL of Ferrozine™ reagent (10 mM Ferrozine™ in 40 mM HCl:20 mM ferric chloride:0.03 M acetate buffer pH 3.6 ratio 1:1:10) were added to each well of a 96-well plate. Ascorbic acid (0.1 mg/mL) was used as a reference in the range of 0–2.5 µg to evaluate POE antioxidant activity. The absorption values were measured at 595 nm at room temperature with a microplate reader.

For DPPH assay, scalar volumes of POE were diluted with methanol (final volume 100 µL) in a 96-well microplate and then mixed with 100 µL of freshly prepared DPPH solution (0.25 mg/mL in methanol). After 30 min incubation in the dark at room temperature, the absorbance was read at 490 nm with a microplate reader. Ascorbic acid (0.5 mg/mL) was used as a reference in the range of 0–15 µg to evaluate the radical scavenging activity of POE.

The TP of POE was expressed as milligrams of gallic acid equivalent (GAE) per milliliter of POE, whereas the antioxidant and radical-scavenging activities were expressed as milligrams of ascorbic acid equivalent (AAE) per milliliter of POE.

POE biochemical characterization results were obtained by plotting the POE absorption values on the respective standard curves of the colorimetric assays.

The determinations were repeated in triplicate. Values are reported as mean ± standard deviation (SD).

### 3.3. Cell Line and Experimental Conditions

American Type Culture Collection (ATCC^®^) provided the human HepG2 hepatoma cell line. Cells were grown in a humidified atmosphere with 5% CO_2_ at 37 °C in DMEM supplemented with 10% FBS, 100 µg/mL streptomycin, 100 U/mL penicillin, and 2 mM L-glutamine (complete medium), with a physiological glucose content (5 mM). Trypsin (0.25% trypsin, 0.5 mM EDTA) was used to detach cells at 90% confluence. The following in vitro cell-based experiments were conducted in complete medium under normal glucose (NG, 5 mM D-glucose) or high glucose (HG, 25 mM D-Glucose) conditions in the absence or presence of POE (7 µg GAE/mL).

### 3.4. Cell Viability

The MTT colorimetric assay was used to examine the viability of HepG2 cells. Cells were cultured in 96-well plates (3 × 10^4^ cells/well) for 24 h under NG condition. Subsequently, cells were exposed to NG or HG conditions in the absence or presence of POE (7 µg GAE/mL) for 24 h. The culture medium was then removed and 100 µL of MTT solution (0.5 mg/mL) was added to each well. After incubation in the dark at 37 °C for 1 h, the insoluble formazan crystals were dissolved in 100 µL/well of dimethyl sulfoxide. An iMARK microplate reader (Bio-Rad Laboratories, USA) was used to measure absorbance values at 595 nm. Data were expressed as percentages compared with untreated control cells under NG condition.

### 3.5. Determination of Intracellular Neutral Lipids by Oil Red O (ORO) Assay

HepG2 cells were seeded (6 × 10^4^ cells/well) in 24-well plates overnight and then exposed to NG or HG conditions in the absence or presence of POE (7 µg GAE/mL) for 24 h. After washing with PBS, the cells were fixed in 2% (*v*/*v*) paraformaldehyde for 10 min. 

The fixed cells must be allowed to dry completely after two washes with PBS. For 30 min at 37 °C, neutral lipids were stained with 200 µL/well of ORO working solution (60% in distilled water). Excess dye was washed with distilled water until the water no longer showed a visible pink color. After complete drying of the wells, the stained lipid droplets in the cells were examined and photographed with a Nikon TS-100 microscope equipped with a digital acquisition system (Nikon Digital Sight DS Fi-1; Nikon, Minato-ku, Tokyo, Japan). Finally, cellular lipid accumulation was measured by adding 200 µL/well of isopropanol. Absorption was measured at 490 nm using an iMARK microplate reader (Bio-Rad Laboratories, USA) [34].

### 3.6. Western Blot Assay

HepG2 cells (15 × 10^4^ cells/well) were cultured in 6-well plates for 24 h. The cells were then exposed to NG and HG conditions in the absence or presence of POE (7 µg GAE/mL) for 7, 16, and 24 h. A Laemmli buffer solution containing Tris-HCl (62.5 mM, pH 6.8), 10% (*w*/*v*) SDS, and 25% (*w*/*v*) glycerol was used to lyse the cells. Lysates were centrifuged at 4 °C for 1 min at 12,000× *g*. The total protein concentration of each sample was determined using the BCA protein assay. Then, 30 µg protein from each sample was mixed with 5% (*v*/*v*) β-mercaptoethanol and bromophenol blue and heated at 95 °C for 5 min. Protein samples were electrophoretically separated on 12% or 15% SDS-polyacrylamide gels and blotted onto PVDF membranes (0.45 µm). After a saturation step with a BSA blocking buffer [5% (*w*/*v*) BSA in 0.1% (*v*/*v*) PBS-Tween^®^-20], the membranes were incubated overnight at 4 °C with primary antibodies appropriately diluted in the blocking buffer. The primary antibodies used are listed in Table 3. After three washes in 0.1% (*v*/*v*) PBS-Tween^®^-20 solution, HRP-linked secondary antibodies of goat anti-rabbit IgG (1:10,000) or goat anti-mouse IgG (1:10,000) (Invitrogen, Waltham, MA, USA) were added to each membrane for 1 h at room temperature. After three washes in 0.5% (*v*/*v*) PBS-Tween^®^-20, Clarity Western ECL solution was used to detect protein bands using the Amersham^TM^ 600 Imager imaging system (GE Healthcare Life Science, Pittsburgh, PA, USA). Quantity One (version 4.6.6, Bio-Rad) was used as the instrument for densitometric analysis of protein bands.

### 3.7. Assessment of MMP-2/9 by Gelatin Zymography

Gelatin zymography was used to assess MMP-2 and MMP-9 metalloproteinase (or gelatinase) activity [22,27]. In 24-well plates, cells were seeded at a density of 2×10^5^ cells/well and incubated overnight. Cells were then treated with POE (7 µg GAE/mL) in DMEM medium supplemented with heat-inactivated serum (at 55 °C for 30 min) with high glucose (HG, 25 mM D-Glucose) for 24 h. As controls, untreated HG cells were used. For pellet cell debris, culture supernatants were collected and centrifuged at 9700× *g* for 1 min at 4 °C. The conditioned medium (2.5 µL) was then separated in an 8% polyacrylamide gel containing gelatin (1 mg/mL) under nonreducing conditions. To remove SDS, the gel was washed twice (30 min/each time) in 2.5% (*v*/*v*) Triton X-100 before being incubated at room temperature for 30 min in reaction buffer (50 mM Tris-HCl pH 7.4, 0.2 M NaCl, 5 mM CaCl_2_, 1 mM ZnCl_2_). Overnight incubation was performed in the reaction buffer. Then, the gel was incubated for 1h at room temperature with a solution containing 40% (*v*/*v*) methanol and 10% (*v*/*v*) acetic acid. Following two washes in double-distilled water (10 min each), staining was done with colloidal Coomassie Brillant Blue G-250 (0.05%) dissolved in 1.6% (*v*/*v*) phosphoric acid, 8% (*w*/*v*) ammonium sulfate, and 20% (*v*/*v*) methanol. Gelatinase activities were clear after stain removal, with 1% (*v*/*v*) acetic acid appearing as clear bands on a blue background. A digital scanner was used to acquire zymography images.

### 3.8. Statistical Analysis

Unless otherwise indicated, data are expressed as mean ± standard deviation (SD) of independent experiments.

For cell viability (MTT assay), intracellular lipid accumulation (ORO assay), and gelatinase activity (gelatin zymography) experiments, signals acquired from independent experiments were normalized by centering the mean (i.e., each replicate measurement was divided by the mean of the triplicates in order to compensate for experimental batch fluctuations), and differences were assessed by one-way ANOVA followed by the post-hoc Tukey’s HSD test.

For Western blotting analysis, differences between normalized intensity signals were evaluated by Kruskall–Wallis test followed by Conover’s post hoc test. Statistical differences were defined at *p* ≤ 0.05.

## 4. Conclusions

There are good reasons to state that high glucose levels may have a pro-tumor role. High glucose activates various signaling pathways that cooperate in controlling the behavior of cancer cells contributing to its development and progression.

Here, it was demonstrated that the polyphenol-rich leaf extract of *P. oceanica* (POE) prevents intracellular lipid accumulation and blocks the MAPKs/NF-κB axis, and consequently reduces MMP-2/9 in HG-exposed HepG2 cells, used as an in vitro model of HCC. 

Altered lipid metabolism may affect NF-κB signaling pathway, promoting inflammation and fibrosis and supporting HCC progression. Targeting glucose-induced de novo lipogenesis and NF-κB activity could therefore prove to be an interesting therapeutic target in the prevention and management of HCC.

In light of POE ability to reduce de novo lipogenesis and regulate the NF-κB signaling pathway, we suggest the marine plant *P. oceanica* as a potential dual weapon against HCC progression. To date, some inhibitors of the NF-κB pathway are in various stages of clinical trials [56]. However, a series of drawbacks and side effects of the conventional drugs lead to the continuous search for new, safer, and more effective molecules for the prevention and/or adjuvant therapy of HCC. Scientific research has made great strides in the study of phytochemicals in the treatment of cancer. The safer profile of natural compounds has given new hope for the design of new adjuvant therapeutic approaches aimed at reducing cancer progression by limiting the side effects of conventional therapies. In light of these considerations, this study lays the foundations for further in vitro and/or in vivo investigations to study the marine plant *P. oceanica* in adjuvant cancer therapy.

## Figures and Tables

**Table 1:** Percent composition of constituent polyphenols of the hydroalcoholic extract of *P. oceanica* leaves (POE) compared with total polyphenols.

Polyphenol	Chemical Structure	Percentage (%)
(+) Catechin	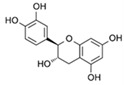	85
Ferulic acid	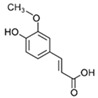	1.7
Epicatechin	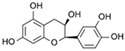	1.4
Chlorogenic acid	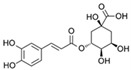	0.6
Gallic acid	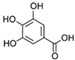	0.4

**Table 2:** Total polyphenols and antioxidant properties of POE.

	Method	Reference Control	POE
Total polyphenols	Folin–Ciocalteau	gallic acid	3.6 ± 0.3 GAE mg/mL
Antioxidant activity	FRAP	ascorbic acid	1.0 ± 0.2 AAE mg/mL
Radical scavenging activity	DPPH	ascorbic acid	10 ± 2.0 AAE mg/mL

**Table 3:** List and specifics of primary antibodies used for Western blot assay.

Primary Antibody	Dilution	Isotype	Source
p-NF-κB	1:1000	Rabbit IgG	Cell Signaling
NF-κB	1:1000	Rabbit IgG	Cell Signaling
IκBα	1:1000	Rabbit IgG	Cell Signaling
p-p38	1:1000	Mouse IgG	Santa Cruz
p38	1:1000	Mouse IgG	Santa Cruz
p-ERK1/2	1:2000	Rabbit IgG	Cell Signaling
ERK1/2	1:1000	Rabbit IgG	Cell Signaling
FASN	1:1000	Rabbit IgG	Cell Signaling
GAPDH	1:1000	Mouse IgG	Invitrogen
α-Tubulin	1:1000	Rabbit IgG	Genetex

## Data Availability

The data presented in this study are available within the article or supplementary material.

## Supplementary Materials

The supporting information can be downloaded at: https://www.mdpi.com/article/10.3390/ijms24065203/s1.
