# Supplementary material for: Ameliorative Effect of Posidonia oceanica on High Glucose-Related Stress in Human Hepatoma HepG2 Cells
Source: Int J Mol Sci. 2023 Mar 8;24(6):5203. doi: 10.3390/ijms24065203 (PMC10048879; doi:10.3390/ijms24065203)

Supplementary Materials

Figure S1. Expression levels of FASN over time in HepG2 cells under normal glucose conditions (NG, 5 mM). Signal quantification was determined by densitometric analysis. Data were reported as the mean of independent experiments, and normalized to the respective expression levels of the α-Tubulin housekeeping protein. Error bars represent standard errors.

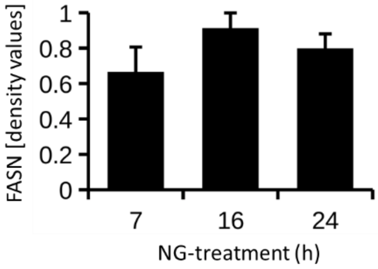

Figure S2. Expression levels over time of FASN in HepG2 cells in the absence or presence of POE (7 μg GAE/mL) under normal glucose conditions (NG, 5 mM). (A) Representative image of Western blot analysis. α-Tubulin (55 kDa) was used as a housekeeping protein in the expression analysis and as the loading control. Quantification of the signals of (B) FASN was determined by densitometric analysis. Data were reported as the mean of independent experiments, and in terms of percentages compared with NG untreated control cells (represented by dashed line). Error bars represent standard errors.

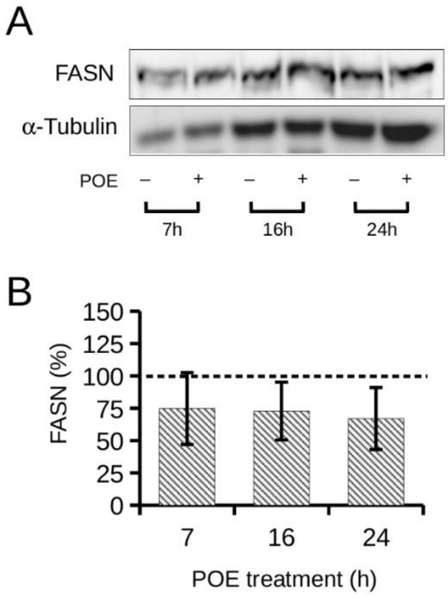

Figure S3. Expression levels over time of protein markers of NF-κB and MAPKs signaling pathways in HepG2 cells under normal glucose conditions (NG, 5 mM). Quantification of the signals of (A) p-NF-κB versus total NF-κB protein, (B) IκBα, (C) p-p38 versus total p38 protein, and (D) p-ERK1/2 versus total ERK1/2 protein was determined by densitometric analysis. Data were reported as the mean of independent experiments, and normalized to the respective expression levels of the GAPDH housekeeping protein. Error bars represent standard errors.

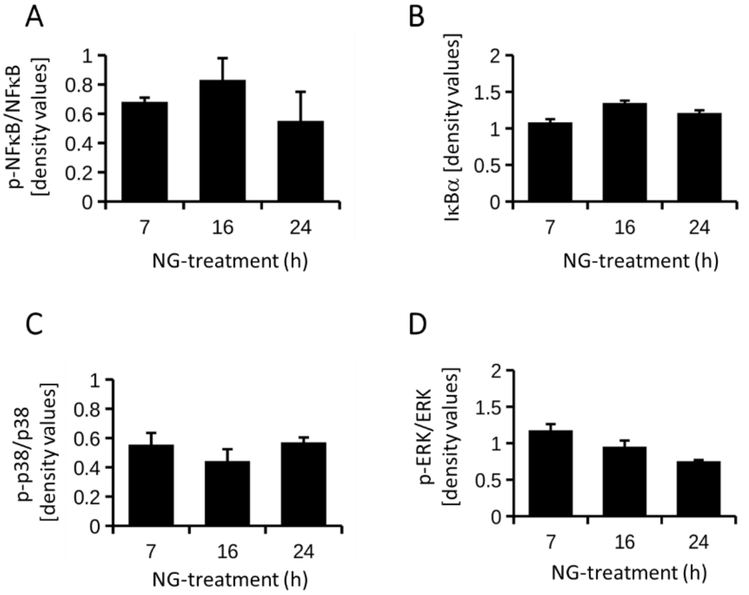

Figure S4. Expression levels over time of protein markers of NF-κB and MAPKs signaling pathways in HepG2 cells in the absence or presence of POE (7 μg GAE/mL) under normal glucose conditions (NG, 5 mM). (A) Representative image of Western blot analysis. GAPDH (37 kDa) was used as a housekeeping protein in all expression analyses and as the loading control. Quantification of the signals of (B) p-NF-κB versus total NF-κB protein, (C) IκBα, (D) p-p38 versus total p38 protein, and (E) p-ERK1/2 versus total ERK1/2 protein was determined by densitometric analysis. Data were reported as the mean of independent experiments, and in terms of percentages compared with NG untreated control cells (represented by dashed line). Error bars represent standard errors. Kruskal-Wallis test.

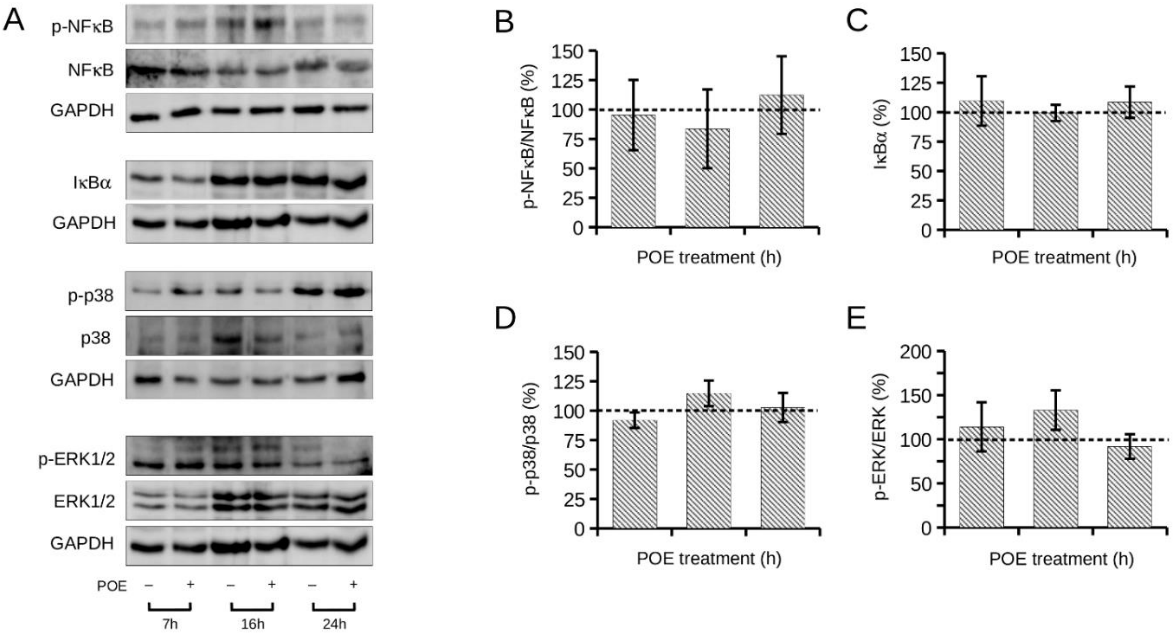

Supplement: Supplementary file 1 [file ijms-24-05203-s001.zip › ijms-2237178-supplementary.pdf]
